# Measures of multiple deprivation and visual field loss in glaucoma clinics in England: lessons from big data

**DOI:** 10.1038/s41433-023-02567-z

**Published:** 2023-05-10

**Authors:** Mehal Rathore, Yusrah Shweikh, Stephen R. Kelly, David P. Crabb

**Affiliations:** 1 Department of Optometry and Visual Sciences, School of Health & Psychological Sciences, City, University of London, London, UK; 2 Sussex Eye Hospital, University Hospitals Sussex NHS Foundation Trust, West Sussex, UK

**Keywords:** Epidemiology, Optic nerve diseases

## Abstract

**Background/Objectives:**

To examine the association between multiple deprivation with late diagnosis and rapid worsening of glaucoma in patients in English hospital eye services (HES).

**Methods:**

602,439 visual fields (VFs) were extracted from five regionally different glaucoma clinics in England. Mean Deviation (MD) worse than −12 dB was used as a surrogate definition for advanced VF loss at diagnosis in patients with ≥2 reliable VF records. MD loss worse than -1 dB per year was used to define rapid VF progression in patients with ≥6 VFs. Patient data were stratified into deciles of the Index of Multiple Deprivation (IMD) from residential postcodes.

**Results:**

There was an association between IMD and advanced VF loss at diagnosis in 44,956 patients with 18% (293/1608) and 11% (771/6929) in the most and least deprived IMD decile, respectively. Age-corrected odds ratio (OR) for having advanced VF loss at entry into HES was 1.42 (95% confidence interval [CI] 1.21–1.67) and 0.75 (95% CI: 0.66–0.85) in the most and least deprived IMD decile respectively (reference = fifth decile). In 15,094 patients with follow up data (median [interquartile range] of 6.9 [4.5, 10.0] years), the proportion having rapid VF progression did not differ across the IMD spectrum.

**Conclusion:**

Large-scale VF data from clinics indicates that glaucoma severity at presentation to English HES is associated with levels of multiple deprivation. We found no evidence to suggest likelihood of having rapid VF progression during follow-up is associated with IMD; this hints at equity of glaucoma care and outcomes once patients are in English HES.

## Introduction

If glaucoma is not detected early enough or if it is not well managed, the then subsequent visual field (VF) loss can adversely affect a patient’s vision-related quality of life. Late presentation of glaucoma, when a patient already has advanced VF loss at diagnosis, is a major risk factor for subsequent loss of vision [1, 2]. Primary open-angle glaucoma, the most common form of the condition, is mostly asymptomatic in its early stages and early detection relies on a person having regular eye examinations. The vast majority of glaucoma referrals to hospital eye services (HES) in England are generated by examinations at community optometrists [3–5]. Yet, people with lower socioeconomic status, lower educational attainment, and less awareness of eye disease are probably less likely to engage with primary healthcare services for this “opportunistic” eye health screening, recommended to be done at least once every 2 years [6].

Poor socioeconomic status (SES) is related to poorer health. This association is very well established, and it exists even in relatively prosperous parts of the world. Eye health is not an exception to this rule, and this is well described in a recent comprehensive systematic review [7]. The specific association between the late presentation of glaucoma and SES is recognised. For example, more than 20 years ago Fraser and colleagues demonstrated that people exposed to higher levels of multiple deprivation were more likely to have late presentation of glaucoma at HES in England [8]. Further evidence has emerged to corroborate this association in England [9], Scotland [10] and other parts of the world [11, 12]. An up-to-date examination of this association, in a larger number of patients from different parts of England, would be useful and this is one of the main ideas of this study.

Aside from its use in clinical management, an electronic medical record (EMR) allows for the collection of clinical data from large patient populations. In turn, these data can be used for identifying trends in disease worsening and treatment response on a large scale [13]. Linking these ‘big data’ to other public health data like those developed for measuring a person’s SES opens up interesting areas of research and our study serves as an example of this. One such data set is the index of multiple deprivation (IMD), which is the most widely used English measure of SES at the small area (postcode) level, aimed at assisting policymakers. IMD is based on seven metrics: income, employment, education, health, crime, living environment, and access to housing and services [14]. IMD data has been widely used previously to show that deprivation is a significant factor in the prevalence or presentation of a number of specific eye conditions [15–17]

The purpose of this study is to present large-scale multicentre data on a measure of glaucoma severity at presentation to HES in England and to see how this varies across the SES spectrum as measured by IMD. In addition, nothing is known about the effect of multiple deprivation on glaucoma management outcomes in patients receiving long-term treatment from HES in England. We, therefore, test the novel hypothesis that rates of VF progression (as a measure of glaucoma worsening) in diagnosed patients in HES in England vary with a patient’s level of multiple deprivation.

## Methods

This study is a retrospective analysis of large-scale data extracted from EMRs as described elsewhere [13, 18, 19]. In short, data recorded between April 2000 and March 2015 were extracted and anonymised from the Medisoft EMR (Medisoft, Leeds, UK) at five regionally different National Health Service (NHS) Hospital Trust glaucoma clinics in England. These clinics provide secondary eye care to people referred by primary care providers, mainly community optometrists. Each centre is the only NHS provider of glaucoma care to their local population, and relatively few patients switch between providers or access care privately. The original data extraction was commissioned by the Healthcare Quality Improvement Partnership overseen by the Royal College of Ophthalmologists as the National Ophthalmology Database Audit provider. Data were securely held on a university database. The study adhered to the Declaration of Helsinki and the General Data Protection Regulation of the European Union. Subsequent analyses of the data were approved by a research ethics committee of City, University of London with one condition being the five NHS centres were anonymised.

The original data extraction yielded 602,439 separate VF records from 73,994 people. These data also included age (years) and a minimum data set of other clinical records. At the source, the EMR includes data on residential addresses as standard. Each postcode was identified and allocated to the IMD score for that area (LSOA; lower layer super output area) based on the English Indices of Deprivation 2015 (https://www.gov.uk/government/statistics/english-indices-of-deprivation-2015). Notably, the LSOA conversion was done at the source to avoid the transfer of patient-identifiable data, and postcodes were never extracted. The English IMD 2015 uses the LSOAs defined in the 2011 census, with the evaluation of deprivation being primarily based on data taken from 2012 to 2013. Merging and matching the VF data to the available IMD data yielded complete records from 69 587 people.

Only VFs recorded by adults (≥18 years) on the Humphrey Field Analyzer (HFA; Carl Zeiss Meditec, California, USA) using a Goldmann size III stimulus with a 24-2 test pattern acquired with the Swedish Interactive Testing Algorithm (SITA Fast or SITA standard) were included. We excluded all unreliable VF records using the criterion of a percentage of false-positive errors ≥15%. No exclusion criteria were applied based on fixation losses or false-negative errors as recommended by previous research [20, 21].

For testing the hypothesis that presenting at HES with advanced VF loss is associated with SES, we defined as our population people who had had at least two separate visits to the clinic. Therefore, we excluded all those patients that had only one visit or one set of VF records assuming most of these would have been false referrals from primary care. We used HFA mean deviation (MD) as a measure of the overall severity of a VF loss. MD is calculated relative to visually healthy peers, with more negative values indicating greater VF loss. The stage of VF loss at presentation was estimated by MD in the worse eye (the one with the more negative MD) at the second VF examination. The second VF was used to mitigate the bias of the perimetry learning effect [13, 22]. We chose the worse eye as a surrogate of the most ‘detectable’ level of VF loss at the stage of case finding in primary care. Patients with MD worse than −12 dB in this eye were defined as having *advanced VF loss at presentation*. This VF criterion has been widely used, including in health economic investigations of service delivery of glaucoma [13, 23].

For testing the hypothesis that patients in glaucoma clinics have rapid VF progression associated with lower SES, we then defined our population as patients with at least six visits to the clinic. These patients were simply a subset of those from the previous cross-sectional analysis. The speed (rate) of VF loss in these patients in clinics was determined by using ordinary linear regression of MD against the time of follow-up (dB/year); a standard method that is widely used [13, 20]. The first VF examination in each series was removed to account for the perimetry learning effect. We used all the patient’s follow-up data meaning that the rate of VF loss could be estimated more precisely, from more than six records and over longer periods where available. Again, we only used one eye per patient, the one with the more negative (worse) MD at the second (baseline) VF examination. Patients with a rate of loss worse than −1 dB/year in this eye were defined as having *rapid VF progression*; this criterion has been widely used in previous studies [24–26].

Patients were stratified into their IMD deciles one to ten as described on https://www.gov.uk/government/statistics/english-indices-of-deprivation-2015. Decile 1 (IMD 1) corresponds to the most deprived group. We calculated summary statistics for MD (dB) at presentation and MD (dB) loss per year for patients stratified by decile of IMD. We also calculated the proportion of patients with the outcome of interest, that is having advanced VF loss at presentation and having rapid VF progression for each decile. We used age-corrected logistic regression to calculate an odds ratio (OR) for having each outcome of interest across all the deciles (the spectrum of SES) using the fifth IMD decile set as the reference group, as has been done previously [27]. Under the null hypothesis that the IMD is not associated with the outcomes of interest, we would expect the age-corrected ORs not to be statistically different from one. All statistical analyses were done using R statistical software (R Foundation for Statistical Computing, Vienna, Austria).

## Results

**Fig. 1:**
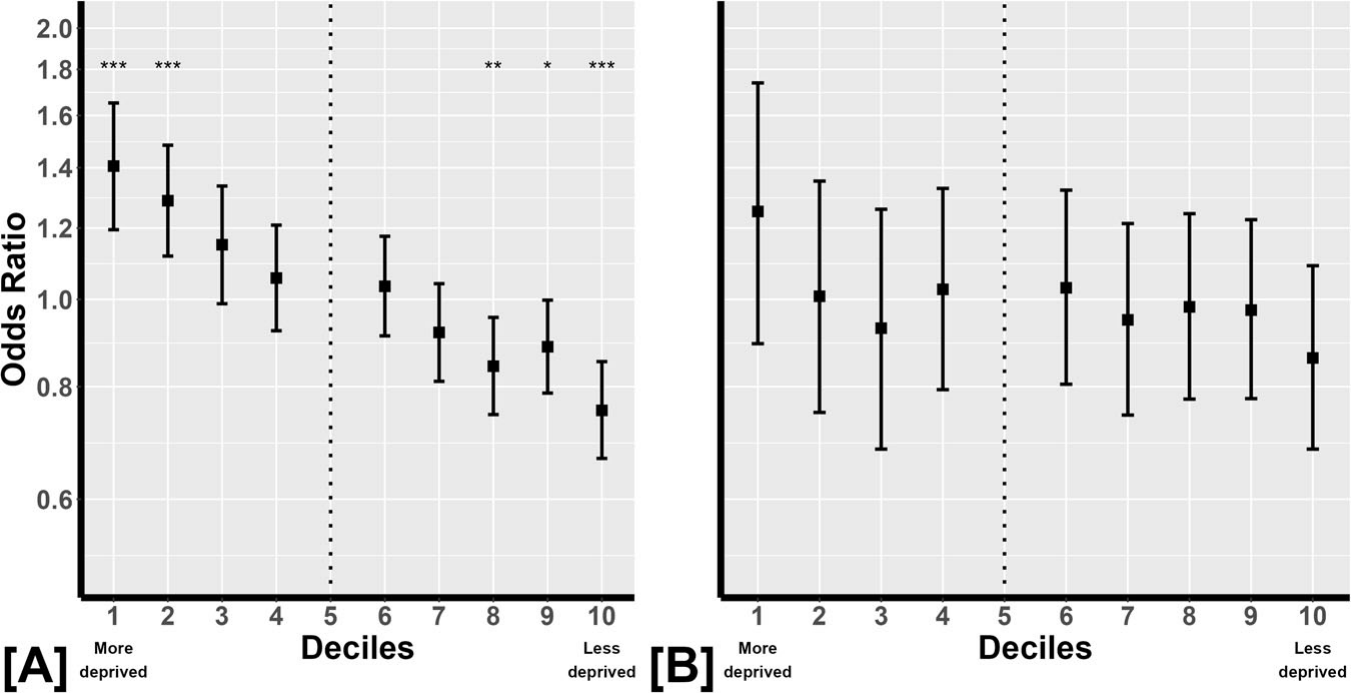
Odds ratio. **A** graphically shows the age-corrected odds ratio (OR) and corresponding 95% confidence intervals (bars) for having advanced visual field (VF) loss at presentation to clinic for each index of multiple deprivation (IMD) decile (The most deprived being IMD decile 1 and the least deprived being IMD decile 10). The asterisks indicate level of statistical significance (<0.05, <0.01 and <0.001 for *, ** and **, respectively) for the age-corrected OR being different from the value for reference fifth decile (dotted line). **B** is formatted in exactly the same way but shows the age-corrected OR and corresponding 95% confidence intervals (bars) for having rapid VF progression whilst followed in clinic for each IMD decile. In this case none of the ORs are statistically different relative to the fifth decile (dotted line). Note the ORs are plotted on an appropriate log scale on the vertical axis.

The inclusion criteria for the cross-sectional analysis yielded 44,956 patients. Data for these patients as stratified by IMD decile are given in Table 1. Median age (years) is strikingly similar across all IMD deciles but the median MD at presentation clearly varies across the IMD deciles, being worse in patients exposed to more deprivation. For example, the difference in median MD between those in the most and least deprived deciles is >1.5 dB; the magnitude of the effect is noteworthy. Moreover, 18% (293/1608) of patients had advanced VF loss at presentation in the most deprived IMD decile whereas this value was 11% (771/6929) for those in the least deprived IMD decile. There was a clear pattern of association between IMD (across all deciles) and the likelihood of having advanced VF loss at diagnosis (Fig. 1A). For example, the age-corrected OR for having *advanced VF loss* at entry into HES was 1.41 (95% confidence interval [CI] 1.20 to 1.62) and 0.75 (95% CI: 0.67 to 0.85) in the most and least deprived IMD decile respectively (relative to the fifth decile in all cases).

**Table 1:** Description of cross-sectional data - advanced visual field loss at presentation to clinic.

1		Median (IQR)	Median (IQR)	% (*n*)	OR (95% CI)	*p* value
IMD Decile	*n*	age (years)	MD (dB)	advanced VF loss (dB)		
1	1608	68 (57, 77)	−4.32 (–9.00, –1.88)	18 (293)	1.41 (1.20–1.62)	<0.001
2	2708	68 (56, 77)	−3.88 (–8.56, –1.62)	17 (454)	1.29 (1.12–1.48)	<0.001
3	2306	69 (57, 77)	−3.91 (–8.17, −1.73)	16 (361)	1.15 (0.99–1.34)	0.07
4	3629	69 (59, 77)	−3.67 (−7.80, −1.50)	15 (537)	1.06 (0.92–1.21)	0.43
5	3361	69 (60, 77)	−3.24 (−7.39, −1.28)	14 (480)	1	–
6	4768	69 (60, 78)	−3.29 (−7.52, −1.26)	15 (704)	1.03 (0.91–1.18)	0.60
7	5788	69 (59, 77)	−3.13 (−7.07, –1.15)	13 (762)	0.92 (0.81–1.04)	0.18
8	6246	69 (60, 77)	−2.89 (−6.50, −1.06)	12 (769)	0.84 (0.74–0.95)	0.007
9	7613	69 (60, 77)	−3.00 (−6.97, −1.09)	13 (988)	0.89 (0.79–1.00)	0.045
10	6929	69 (60, 77)	−2.77 (−6.29, −1.00)	11 (771)	0.75 (0.67–0.85)	<0.001
Total	44,956	69 (59, 77)	−3.19 (−7.19, −1.22)	14 (6119)		

*VF* visual field, *n* number of patients, *y* years, *MD* mean deviation, *IMD* index of multiple deprivation, *IQR* interquartile range, *OR* age-corrected odds ratio, *CI* confidence interval.

Inclusion criteria for the longitudinal (follow-up) analysis yielded 15,094 patients. These data were rich in length of follow-up (median [interquartile range] of 6.9 [4.5,10.0] years). So, for example, approximately one-quarter of these patients had at least 10 years of follow-up data available for analysis giving us confidence in the estimates of the rates of MD loss. Data for these patients as stratified by IMD decile are given in Table 2. As with the cross-sectional sample, median age (years) is similar across all IMD deciles. The median rate of VF loss appears to be similar across all IMD deciles and this is noteworthy. For example, the median rate of VF loss for those in the most (−0.16 dB/year) and least (−0.18 dB/year) deprived deciles were virtually the same. Whilst patients as a group in IMD 1 (most deprived) clearly have worse VF loss at baseline they do not seem to be progressing more quickly, on average, than those in IMD 10 (least deprived). In fact, there was no evidence of an association between IMD and rapid VF progression at all (Fig. 1B). The proportion of patients with the attribute of having rapid VF progression was similar across the deciles. Moreover, even at the extremes of the SES spectrum, the age-corrected OR of 1.25 (95% CI: 0.89 to 1.74) and 0.86 (95% CI: 0.68 to 1.09) in the most and least deprived IMD deciles, respectively, do not significantly differ from the reference value of one (*p* = 0.19 and *p* = 0.21).

**Table 2:** Description of follow-up data - rapid visual field progression whilst followed in clinic.

		Median (IQR)	Median (IQR)	Median (IQR)	Median (IQR)	% (*n*)	OR (95% CI)	*p*
IMD Decile	*n*	age (y)	No. VFs	Follow-up (y)	MD loss (dB/y)	rapid VF loss		
1	488	68 (58, 78)	8 (6, 11)	7.26 (4.62, 10.48)	−0.16 (–0.56, 0.05)	0.13 (63)	1.25 (0.89–1.74)	0.19
2	796	68 (60, 76)	7 (6, 10)	6.91 (4.65, 9.92)	−0.15 (–0.54, 0.10)	0.11 (89)	1.01 (0.75–1.35)	0.96
3	745	69 (59, 76)	8 (6, 11)	6.93 (4.39, 10.23)	−0.16 (–0.52, 0.07)	0.10 (78)	0.93 (0.68–1.26)	0.64
4	1345	68 (60, 76)	8 (6, 11)	7.06 (4.53, 10.35)	−0.20 (–0.55, 0.04)	0.11 (152)	1.03 (0.79–1.33)	0.85
5	1104	69 (60, 76)	7 (6, 10)	7.04 (4.63, 9.90)	−0.18 (–0.50, 0.05)	0.11 (126)	1	–
6	1562	68 (60, 76)	7 (6, 10)	6.85 (4.53, 9.88)	−0.17 (–0.52, 0.03)	0.12 (180)	1.03 (0.80–1.32)	0.81
7	1850	68 (59, 75)	7 (6, 10)	6.75 (4.44, 9.79)	–0.16 (–0.46, 0.07)	0.10 (188)	0.95 (0.74–1.21)	0.68
8	2118	68 (60, 75)	7 (6, 10)	6.80 (4.43, 9.77)	–0.14 (–0.50, 0.06)	0.11 (226)	0.98 (0.78–1.25)	0.87
9	2591	69 (60, 76)	7 (6, 11)	6.78 (4.52, 9.81)	–0.18 (–0.54, 0.03)	0.11 (283)	0.97 (0.78–1.23)	0.82
10	2495	68 (60, 75)	7 (6, 10)	6.78 (4.42, 10.02)	–0.18 (–0.50, 0.05)	0.09 (236)	0.86 (0.68–1.09)	0.21
Total	15,094	68 (60, 75)	7 (6, 10)	6.86 (4.49, 9.97)	–0.17 (–0.51, 0.05)	0.11 (1621)		

*VF* visual field, *n* number of patients, *y* years, *MD* mean deviation, *IMD* index of multiple deprivation, *IQR* interquartile range, *OR* age-corrected odds ratio, *CI* confidence interval.

## Discussion

By using retrospectively collected data from 44,956 patients from five regionally different HES glaucoma clinics across England we show a clear association between late presentation of glaucoma and SES. More specifically, we show the likelihood of having advanced VF loss at the first visit to a glaucoma clinic to be much greater in people living in areas with higher multiple deprivation indices compared to peers from areas with lower multiple deprivation. We also demonstrated rapid worsening of vision (using a surrogate of the rapid rate of VF progression) in patients managed by HES across England is not associated with a patient’s level of multiple deprivation.

Our results provide new knowledge on the relationship between a person’s estimated level of multiple deprivation and their glaucoma patient journey in England. First, we have confirmed the reported link between the late presentation of glaucoma and deprivation; this was important because previous UK studies had small sample sizes confined to a particular geographical region [8–10]. Second, we present a novel finding using follow-up data from 15,904 glaucoma patients being managed within English HES where we found no evidence of an association between rapid VF worsening and patients’ IMD. More precisely a patient from a more deprived area, compared to a peer from a less deprived area, is not more likely to lose VF rapidly in HES glaucoma clinics. Importantly, these data hint at an equity of glaucoma management and clinical care once patients are in HES in England. This finding is both novel and reassuring; it contradicts what others have found about the relationship between glaucoma outcomes and SES, albeit in different health systems in different countries [28, 29].

Our results should be discussed in the context of other published studies considering the relationship between SES (multiple deprivation) and glaucoma detection and management, specifically in England. Fraser et al. first reported evidence on the relationship between the late presentation of glaucoma and various indicators of deprivation in a case-control study across three centres [8]. Sukumar and colleagues also showed the association between levels of deprivation and presenting VF loss in a retrospective single-centre study of 113 patients [9]. This general finding was corroborated in a sample of 126 patients in Scotland [10] and in another more recent study of 472 consecutive newly referred patients in Scotland [30]. It has also been shown that people with self-reported glaucoma had higher levels of deprivation and lower income compared to other individuals in the UK Biobank cohort [31]. There are examples of evidence for the association between the late presentation of glaucoma and measures of SES in other countries, but the effect is likely heavily dependent on the health system used and the general level of deprivation [11, 12, 32, 33]. The interested reader is directed to a recent systematic review by Lane and colleagues, which concluded with a call to policymakers to act on untangling the complex link between deprivation and late presentation of eye conditions [7]. Most recently, King and colleagues reported on the effects of SES (as measured by IMD) on baseline values and outcomes from the Treatment of Advanced Glaucoma Study (TAGS) randomised controlled trial [34] (TAGS was primarily designed to determine whether primary medicine or primary surgery is more effective for patients presenting with advanced glaucoma). In 453 patients presenting with advanced VF loss, IMD was correlated with poorer self-reported visual function, poorer MD and visits to a community optometrist in the years preceding diagnosis. Interestingly the TAGS investigators found no evidence that IMD was associated with the effect of the success of treatment at 24 months which supports our finding that VF worsening may not be influenced by SES.

Explanations for the associations we report from our data are largely beyond this discussion, but we briefly speculate on some here. In England, late glaucoma presentation can be explained by delays at the patient level (e.g., less frequent attendance to optometrists) or delays at the healthcare provider level [6]. Interestingly, results from a mapping study of optometrists in an area of Northern England support the theory that people with lower SES present late due to limited access to primary eye care services [35]. In contrast, reasons for delayed access to eye healthcare have been recently shown, in Northern Ireland, not to be linked to low income, for example [36]. Moreover, others have shown no robust evidence of a direct association between SES and access to eye health services in the UK [37]. Reasons underpinning poor access to regular eye testing with optometrists in England are likely complex and may include poor knowledge of eye health, fear, inertia, perceived costs, limited mobility, and access [38, 39]. These explanations and others for the late presentation of advanced glaucoma have been recently reviewed [2].

An obvious strength of our study is the vast number of patient records used to estimate our measures of interest. Moreover, data was multicentre from regionally different clinics across England, although the sampling was not done systematically. Another strength of our study was maintaining the anonymity of the patient records; IMD requires postcode information, but the data linkage was undertaken at source within the HES EMR to avoid the transfer of patient-identifiable data. We also used established methods for assessing VF data and our criterion for late presentation and rapid VF loss have been widely used in other studies. Our chosen method of analysis sensibly corrected for age too, which is known to be associated with late presentation of VF loss and faster VF progression [40].

Our study had several limitations because it is retrospective and lacked information about the complete diagnoses of patients. We cannot be certain that a patient’s first VF is truly their first one after diagnosis because a person may have simply moved, for example. Yet with these large numbers of VF records, we expect any effect of these transfers to be small. Moreover, our surrogate for advanced VF loss, or rapid VF loss, could be affected by a patient having concomitant eye disease. Lack of clinical information restricts the level of certainty about the underlying diagnosis. Again, the size of our sample helps mitigate against this, and only including patients with VF loss being regularly seen in HES glaucoma clinics, should exclude most cases of non-glaucoma diagnosis. We could not differentiate between different types of glaucoma in this study; interestingly, for example, it has been recently shown that deprivation is an important risk factor for patients presenting with acute angle closure glaucoma [41].

Ultimately, we have provided a useful assessment of issues around multiple deprivation and VF loss in glaucoma clinics in England, but the results are still not entirely current being based on data extracted in 2015. Our most novel finding is observing no association between IMD and rapid rates of VF loss in patients in glaucoma clinics in HES. However, as always, the absence of evidence of an association is not evidence of the absence of an effect. Nevertheless, the data we use to illustrate this non-association is robust being based on 15,094 patients with a median of seven HES visits with, remarkably, about one-quarter of patients followed for a decade or more. Moreover, average rates of VF loss are expected to be greater in those eyes with more VF loss at diagnosis – this is a well-established association [40, 42]. So, it is surprising that those with more deprivation and more VF loss did not, on average, have worse rates of loss than those with less deprivation and less VF loss; we think this adds to the evidence of no association between rapid VF progression and multiple deprivation. Nonetheless, our results ought to be replicated in an observational cohort study facilitated by an EMR and this should be the subject of future work. Such a study could also quantify real vision loss in people who received a glaucoma diagnosis later than others, in part due to where they live and their IMD. Such data might support the idea of an intervention, perhaps screening for eye disease in selected groups (not the whole adult population), something that was considered a reasonable idea in a study done some time ago and has recently been revisited [23, 43]. Such future work ought to consider the well-reported complexity of estimating deprivation and SES [44]. In fact, future studies ought to aim to untangle all the causes of people presenting at HES with advanced VF loss.

In summary, the likelihood of a patient presenting to glaucoma clinics in England with advanced VF loss is likely influenced by their level of multiple deprivation. Yet there is no evidence to suggest that their level of multiple deprivation is related to their glaucoma worsening whilst being managed by HES; this finding is both novel and reassuring. Our study and similar ones [13, 40] should act as motivation for others to use routinely collected data in eye clinics to assess aspects of eye health service delivery.

## Summary

### What was known before

- Socioeconomic status adversely influences health-seeking behaviour and eye health outcomes.
- Index of multiple deprivation is a surrogate measure of socioeconomic status in England.
- Advanced visual field (VF) loss at the point of glaucoma diagnosis (presentation) and rapid visual field loss during clinical follow-up are both related to adverse visual outcomes in people with glaucoma.

### What this study adds

- Confirmation of the association between multiple deprivation and late presentation of glaucoma in a large dataset. Patients living in areas with high multiple deprivation are significantly more likely to present with advanced VF loss in hospital eye services (HES) compared to peers from areas with lower multiple deprivation.
- No evidence to support the idea that multiple deprivation is associated with an increased risk of rapid VF progression during follow-up. The lack of a relationship between fast disease progression and socioeconomic status hints at equity in terms of glaucoma management in English HES.
